# Usability of Augmented Reality Technology in Situational Telementorship for Managing Clinical Scenarios: Quasi-Experimental Study

**DOI:** 10.2196/47228

**Published:** 2023-10-02

**Authors:** Dung T Bui, Tony Barnett, Ha Hoang, Winyu Chinthammit

**Affiliations:** 1 Centre for Rural Health, School of Health Sciences, College of Health and Medicine, University of Tasmania Launceston Australia; 2 Human Interface Technology Laboratory, School of Information and Communication Technology, College of Sciences and Engineering, University of Tasmania Launceston Australia

**Keywords:** augmented reality, mentorship, patient simulation, patient care management, quasi-experimental study, telehealth

## Abstract

**Background:**

Telementorship provides a way to maintain the professional skills of isolated rural health care workers. The incorporation of augmented reality (AR) technology into telementoring systems could be used to mentor health care professionals remotely under different clinical situations.

**Objective:**

This study aims to evaluate the usability of AR technology in telementorship for managing clinical scenarios in a simulation laboratory.

**Methods:**

This study used a quasi-experimental design. Experienced health professionals and novice health practitioners were recruited for the roles of mentors and mentees, respectively, and then trained in the use of the AR setup. In the experiment, each mentee wearing an AR headset was asked to respond to 4 different clinical scenarios: acute coronary syndrome (ACS), acute myocardial infarction (AMI), pneumonia severe reaction to antibiotics (PSRA), and hypoglycemic emergency (HE). Their mentor used a laptop to provide remote guidance, following the treatment protocols developed for each scenario. Rating scales were used to measure the AR’s usability, mentorship effectiveness, and mentees’ self-confidence and skill performance.

**Results:**

A total of 4 mentors and 15 mentees participated in this study. Mentors and mentees were positive about using the AR technology, despite some technical issues and the time required to become familiar with the technology. The positive experience of telementorship was highlighted (mean 4.8, SD 0.414 for mentees and mean of 4.25, SD 0.5 for mentors on the 5-point Likert scale). Mentees’ confidence in managing each of the 4 scenarios improved after telementoring (*P*=.001 for the ACS, AMI, and PSRA scenarios and *P*=.002 for the HE scenario). Mentees’ individual skill performance rates ranged from 98% in the ACS scenario to 97% in the AMI, PSRA, and HE scenarios.

**Conclusions:**

This study provides evidence about the usability of AR technology in telementorship for managing clinical scenarios. The findings suggest the potential for this technology to be used to support health workers in real-world clinical environments and point to new directions of research.

## Introduction

### Background

Many rural and remote areas experience a shortage of care professionals [1]. The lack of professional support contributes to these shortages [2]. Professional support refers to activities that create an environment where personal and professional growth may occur [3] and is an important factor in attracting and retaining health professionals in rural and remote areas [4-11]. Professional support, although emphasized in strategies that aim to address rural health workforce maldistribution [9-11], can be difficult to provide because of the lack of on-site expertise.

The use of telementorship to provide professional support and overcome the geographical barrier of distance has increased. Through telementorship, a medical expert can provide instructions remotely to a novice practitioner at the treatment site in real time [12]. Advanced telecommunication technologies may enhance the effectiveness of telementorship as they support a higher level of information exchange and enhance the sense of the mentor being present with the mentee despite being separated by distance.

Augmented reality (AR) is an immersive experience in which the real world is enhanced by computer-generated, 3D content tied to specific locations or activity tasks [13-15]. The beneficial outcomes of the incorporation of AR technology into telementoring systems in health care environments have been reported globally [16]. They included the reduction in skill errors and focus shifts, the improvement in task completion time and task accuracy, and positive feedback from relevant users. The advantages of this technology make it possible to address the challenges of providing professional support by implementing AR technology in situational telementoring relationships [17].

Very few studies have assessed the application of AR technology in which a mentor guides a mentee to manage complex clinical scenarios. This study aimed to address this gap.

### Aim and Objectives

This study aimed to evaluate the usability of AR technology in telementorship for managing clinical scenarios in a simulation laboratory. The objectives of this study were as follows:

- Assess mentors’ and mentees’ perceptions of the usability and effectiveness of AR technology for telementorship
- Evaluate changes in mentees’ self-confidence and skill performance in the management of clinical scenarios when mentored using AR technology.

## Methods

### Overview

A pragmatic quasi-experimental design was used in this study. A total of 4 mentors and 15 mentees were included in this study. The study protocol was previously published [18] and provides details of the study methodology, including the study setting, participant recruitment, selection of clinical scenarios, experimental procedure, outcome measures, and data collection and analysis.

### AR Telementoring Setup

**Figure 1:**
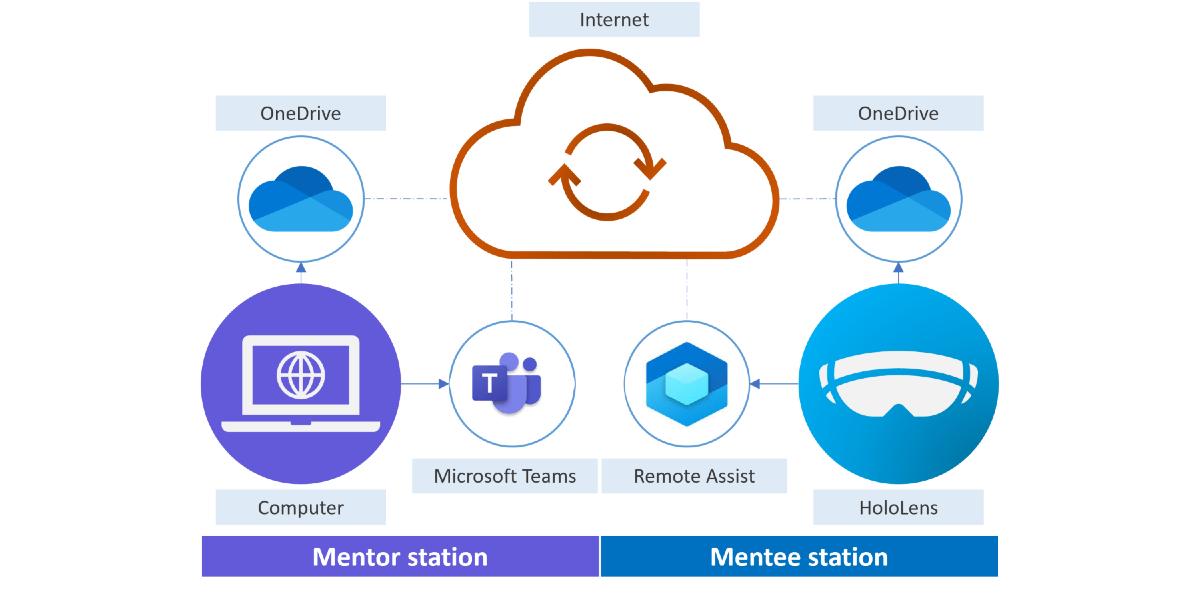
Configuration schematic of an augmented reality telementoring setup.

The AR telementoring setup comprises a mentor station and a mentee station, as illustrated in Figure 1.

The mentor station had a Dell Latitude 5490 laptop. The laptop had a screen size of 14 inches, a screen resolution of 1920×1080 pixels, processor type Intel Core i5-8350U, RAM of 16 GB, and Windows 10. The laptop was connected to a touchscreen, a computer mouse to facilitate annotation, and a headset with ear pads and a noise-canceling microphone to block out ambient noise. The Microsoft Teams software [19] (hereinafter Teams) was installed on the laptop.

The mentee station had a Microsoft HoloLens version 2 (hereinafter HoloLens). The device was an untethered head-worn holographic computer that allowed bidirectional telecommunication via video, voice, and AR or mixed reality composites. It ran using a Window Holographic operating system based on Windows 10. The visor could be flipped up or down, thereby engaging or disengaging the AR or mixed reality content. The HoloLens was also equipped with an adjustable, cushioned inner headband and overhead strap, making it relatively stable and comfortable to wear [20]. The Dynamics 365 Remote Assist software (hereinafter Remote Assist) [21] was installed on the device.

In the study experiments, the laptop and HoloLens were connected to the University of Tasmania’s wireless network.

### Clinical Scenarios

A total of 4 clinical scenarios were selected from 20 patient cases that make up the nursing education scenarios [22]. Following the situational telementorship framework [17], selection criteria were developed to identify scenarios that had a high level of acuity and were likely to place a high demand on the local novice practitioner (the mentee) to manage the patient. The selected scenarios were acute coronary syndrome (ACS), acute myocardial infarction (AMI), pneumonia severe reaction to antibiotics (PSRA), and hypoglycemic emergency (HE). The scenario scripts were reviewed by 2 paramedics, 3 experienced registered nurses, and clinicians and then revised in accordance with the current national protocols of Advanced Life Support [23] and Ambulance Tasmania Clinical Practice Guidelines [24].

**Figure 2:**
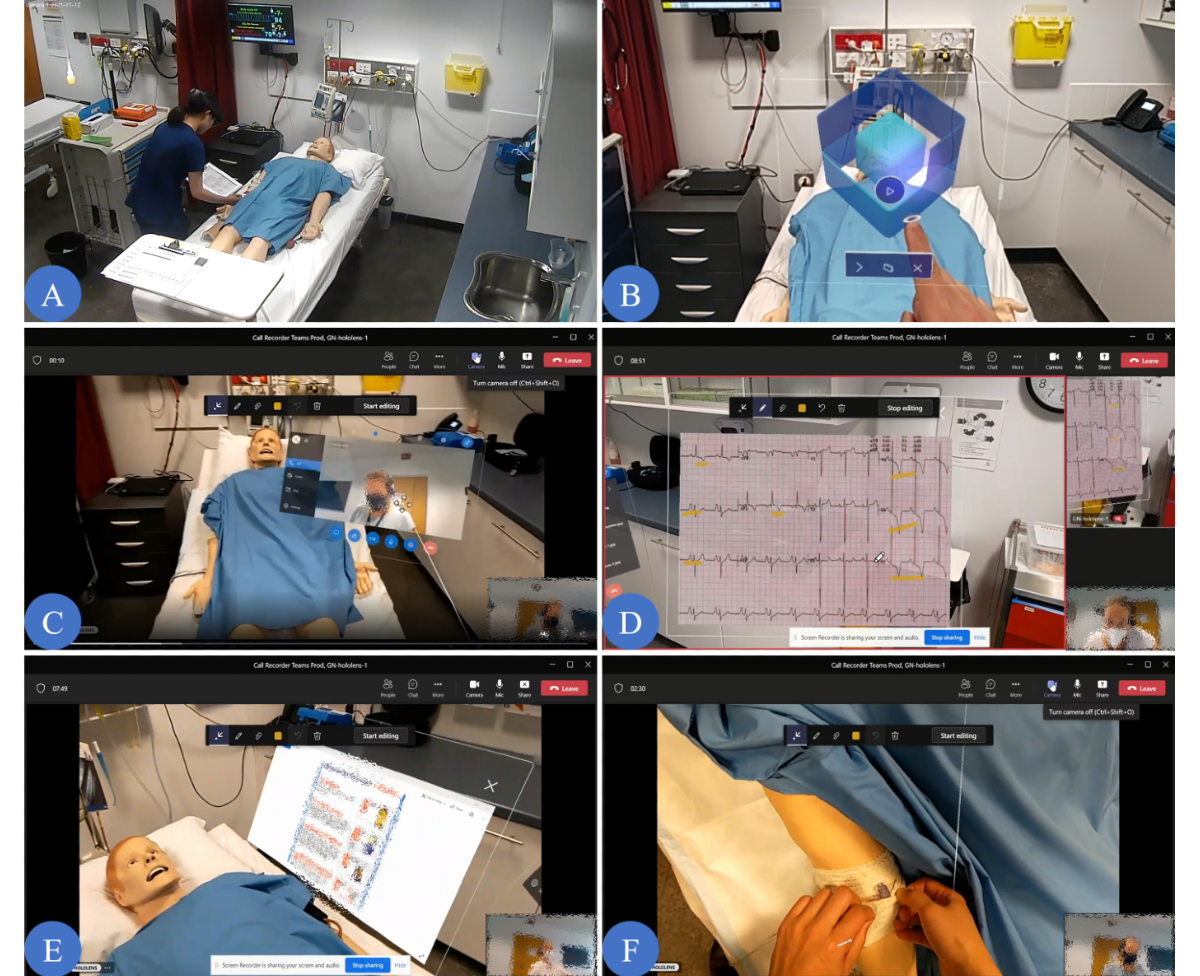
Key moments in a clinical scenario. (A) Initial patient assessment: the mentee assesses the patient’s condition and identifies that they require expert assistance. (B) Initiating remote assistance: the mentee dons the HoloLens and activates the Remote Assist app. (C) Engaging the mentor: the mentee calls the mentor and shares what they see and hear via the device. (D) Mentor reviews the situation: the mentee accesses an electrocardiogram (ECG) from the simulated patient database in the pneumonia severe reaction to antibiotics scenario, for example, via the app, and shares it with the mentor. The mentor then annotates the image to point out abnormal signals in the ECG. (E) Mentor’s advice and instruction: the mentor inserts an assessment instruction into the mentee’s virtual visual space. The mentee then uses this to help assess the patient. (F) Continuation of the telementorship: the mentor guides the mentee on how to manage the patient’s condition when required and observes their performance captured via HoloLens.

All 4 scenarios were scripted to consist of 6 “key moments” representing a sequence of tasks important to the use of the technology in telementorship (Figure 2).

The 7 core features of the AR technology identified as important for remote assistance were incorporated in each scenario script (Textbox 1). An important feature of AR is annotation. This allows images and symbols to be created, transmitted directly into the mentee’s field of view, and anchored to relevant areas of the operating field [25,26]. Studies have shown that the effectiveness of overlaying mentor guidance directly onto the mentee’s view of the operating field resulted in avoiding focus shifts and improving mentee accuracy, compared with conventional telementoring systems [25,27]. As such, opportunities for using annotation by the mentor to guide the mentee was built into all scenarios. The use of wearable technology on AR systems is practical, as the operating clinicians can retrieve information and interact with imaging immediately and intuitively, without having to touch another object or remove sterile gloves [28,29].

**Core features of the augmented reality (AR) technology for remote assistance.**

**Features and explanations**

- Live stream
  Mentees can share their real-time view with mentors in remote locations to obtain the help and guidance [30].
- Hands free
  Mentees can keep both the hands free with head-wearable AR devices to work on their tasks during real-time collaboration with the mentor [31].
- Voice commands
  Mentees and mentors can use voice commands to navigate all features of the AR device, even in a loud, industrial environment [32].
- Recording
  Mentees and mentors can record the call and take screenshots to use for future reference [33].
- Annotation
  Mentees and mentors can use drawings and arrows to refer to specific parts of a machine or asset [33]. These annotations are anchored in the mentee’s visual space.
- Reference insertion
  Mentors can insert reference images, schematics, and other helpful information in the mentee’s field of view [33], so that they can refer to the schematic while working.
- Information storage
  Mentees and mentors can pull in work order information stored in the AR device and call the resource assigned to support them [34].

### Participants

Two groups of participants for the study were mentors and mentees.

Eligible mentors were experienced health professionals, such as medical physicians, registered nurses, or paramedics who were familiar with the clinical scenarios selected in this study. Eligible mentees were health practitioners or soon-to-be registered practitioners, such as registered nurses or paramedics, who were less experienced than the mentors and less familiar with the clinical scenarios. There were no restrictions on their practical experience or previous use of AR devices. They were compensated for their time to participate in the study with a gift card worth Aus $200 (US $128.12).

The participants were selected using a convenient sampling method. A total of 4 mentors and 15 mentees were enrolled in this study [17]. The mentors and mentees did not know each other before participating in the study. Participation in the study was voluntary. They were asked to complete a consent form before participating in the study and were free to withdraw from the study at any time without consequence. Participants were asked whether they would like to be notified of the results of this study at enrollment. If requested, any publication of the study will be forwarded to them.

### Participant Recruitment

A flyer to recruit participants was placed in public media, including the university website, newspapers, and Facebook pages of the university and professional career groups. Interested participants contacted the researcher (DTB) who determined their eligibility based on the inclusion criteria. Once the selection criteria were met, participants were provided with an information package. Snowball sampling was also used for recruitment. The participants could recommend others to join the study.

### Pilot Experiment

A pilot experiment was conducted with 2 paramedic volunteers as mentees. They were suited to being mentees and piloting the scenarios under remote instruction because they were relatively inexperienced and neither had previously used an AR or a similar device.

The technical conditions and laboratory environments were the same as those used for the simulation of the experiment. A one-to-one instruction session was delivered over 2 hours to train the mentees to use the HoloLens. Each mentee was then asked to manage a randomly selected scenario in the high-fidelity simulation laboratory under the remote guidance of an experienced paramedic as the mentor. Although some difficulty was observed in performing hand gestures to control the features of the HoloLens, the pilot demonstrated that the short-term training approach was feasible for participants to learn how to adequately use the HoloLens and the AR features to receive remote assistance in real time. Additional instruction on hand gestures was added to the training sessions for the mentees in the experiments.

### Experimental Procedure

Each mentee was paired up with 1 mentor to perform all 4 scenarios on the day of the experiment. This allowed the mentor and mentee to debrief after each scenario and develop their relationship throughout the course of the experiment. The sequence of scenarios was random for each mentee. All simulation sessions were video and audio recorded.

### Data Analysis

During the data collection period, the research team numbered each retrieved data set and manually examined each data set for potential concerns. All data were then entered and analyzed using SPSS software (version 23.0; IBM Corp). Charts were used to describe the frequencies of categorical variables (age groups, gender, qualification, etc), and mean and SD were computed for continuous variables (scenario completion time, AR usability, mentorship effectiveness, self-confidence, and skill performance scores). The continuous variables were assessed for distribution using histograms, box-whisker plots, and tests of normality, which confirmed the nonnormal distribution. Therefore, alternative nonparametric tests were used to compare scores, including Mann-Whitney *U* tests for between groups (ie, age, gender, and clinical practice) and Wilcoxon signed rank tests for pre- and postscores. Statistical significance was set at *P*<.05. Manifest content analysis was used to analyze the narrative comments provided by the participants in the survey.

### Data Management

Regarding anonymity and confidentiality, the researchers ensured that the experiment was conducted in a safe and confidential place. The researcher did not discuss one participant with another. All data were deidentified and summated, and pseudonyms were used. The data were stored securely both during and after the completion of the study.

Hard-copy data have been stored in a locked, secure location at the university for 5 years after publication. Electronic data, including recorded videos and images, have been stored in a restricted folder accessible only by the chief investigators and the designated Archives Officer. During the study experiments, the researchers secured data, and the electronic data were password protected. The designated Archives Officer will destroy the data after 5 years. Hard-copy materials will be shredded and recycled, and electronic data will be deleted from the secure servers after 5 years.

### Ethical Considerations

The protocol was approved by the Tasmania Health and Medical Human Research Ethics Committee (project ID: 23343).

## Results

### Participant Characteristics

A total of 4 experienced health practitioners, 2 registered nurses, and 2 paramedics, were recruited to participate as mentors in the study. In total, 15 nursing and paramedic participants were recruited as mentees. This was the first time that all mentees and mentors had used AR technology. The 2 experienced nursing mentees had only worked in aged care and general nursing, with limited or no clinical exposure to the events illustrated by any of the 4 scenarios (Table 1).

**Table 1:** Characteristics of the mentees and mentors.

Characteristics		Mentees (n=15), n (%)	Mentors (n=4), n (%)
**Gender**			
	Man	3 (20)	0 (0)
	Woman	12 (80)	4 (100)
**Age group (years)**			
	<30	10 (67)	0 (0)
	≥30	5 (33)	4 (100)
**Current role**			
	Registered nurse	7 (46)	2 (50)
	Paramedic	1 (7)	2 (50)
	Nursing student	6 (40)	0 (0)
	Paramedic student	1 (7)	0 (0)
**Years of clinical practice**			
	Not in practice	7 (46)	0 (0)
	<1	6 (40)	0 (0)
	1 to 10	2 (14)	1 (25)
	>10	0 (0)	3 (75)
**Practice area**			
	Not in practice	7 (46)	0 (0)
	Prehospital or hospital emergency care	2 (14)	2 (50)
	Clinical educator	0 (0)	2 (50)
	Others	6 (40)	0 (0)
**Qualification**			
	Not yet graduated	7 (46)	0 (0)
	Bachelor’s degree	8 (54)	2 (50)
	Graduate diploma	0 (0)	2 (50)
**Simulation experience**			
	Yes	15 (100)	4 (100)
**AR^a^ technology experience**			
	No experience with wearable AR devices, mobile AR devices and apps, and interfaces for hand and body gesture recognition	15 (100)	4 (100)

^a^AR: augmented reality.

### Scenario Performance

The 4 scenarios were performed a total of 60 times (15 mentees, each with 4 clinical scenarios). Of the 60 times, 59 (98%) were completed following the scripts. One scenario (HE) was interrupted in the last minute because the headwear device became overheated. In total, 57 video recordings (>2120 min in total) were generated and assessed. Three videos were lost owing to technical issues with the 4 cameras mounted in the simulation laboratory.

Each mentee took an average of 37 minutes 7 seconds (SD 3 min 30 s) to complete their 4 scenarios. The AMI scenario had a lower average completion time (average 33 min 10 s, SD 7 min 56 s) than the others: PSRA (average 39 min 16 s, SD 8 min 59 s), HE (average 39 min 7 s, SD 6 min 44 s), and ACS (average 37 min 21 s, SD 7 min 43 s). There were no statistically significant differences in scenario completion time (for all scenarios) between the groups based on age (Mann-Whitney *U* tests; *P*=.66 for ACS, *P*=.71 for AMI, *P*=.46 for PSRA, and *P*=.74 for HE), gender (Mann-Whitney *U* tests; *P*=.74 for ACS, *P*=.47 for AMI, *P*=.11 for PSRA, and *P*=.10 for HE), and years of clinical practice (Mann-Whitney *U* tests; *P*=.32 for ACS, *P*=.73 for AMI, *P*=.99 for PSRA, and *P*=.41 for HE).

### HoloLens Use

Across the 60 clinical scenarios, the HoloLens was used for approximately 31.5 hours, representing approximately 89% of the scenario performance periods (more than 35.3 h). Similar to the average completion time of each scenario, the time of using HoloLens was shortest in the AMI, at an average of 27 minutes 38 seconds (SD 5 min 21 s); followed by an average of 33 minutes 38 seconds (SD 7 min 20 s) in the ACS and an average of 34 minutes 40 seconds (SD 7 min 23 s) in the PSRA; and the longest in the HE scenario, at an average of 36 minutes 34 seconds (SD 7 min 0 s).

All 7 core AR features were applied in the simulation sessions, albeit to varying degrees. All mentees shared their real-time views with the mentors while keeping both hands free to work on their tasks. All HoloLens calls were recorded using Microsoft Teams. Mentees accessed “historic” simulated patient case note information such as 12-lead electrocardiographs and chest x-rays stored in the HoloLens and shared this with the mentors 51 times. Mentors then annotated this shared information 47 times using *draw* and *arrow*, the default annotation tools on Microsoft Teams. Mentors inserted references in the mentees’ view 104 times. A total of 10 different references were inserted, for example, the Glasgow Coma Scale, the 8-rights medication check, and the AMPLE (Allergies, Medications, Past Medical History, Last Meal, and Events Leading to Presentation) approach. Mentees preferred to use hand gestures and rarely used voice commands to navigate Remote Assist or to react to the device.

### AR’s Usability

The AR’s usability scales for mentees (n=15) with 42 items and mentors (n=4) with 36 items were completed after the experiment (Tables S1 and S2 in Multimedia Appendix 1). A 5-point Likert scale, with 1 for “strongly disagree” and 5 for “strongly agree” was used.

Although mentees admitted that the clinical scenarios were challenging, they reported that the HoloLens was easy to use (mean 4.07, SD 0.704), and most mentees (14/15, 93%) were confident using it (mean 3.8, SD 0.775). Approximately half (8/15, 53%) of the mentees felt that they would need technical support occasionally and needed to learn more about the technology before using it in the work environment. The majority (12/15, 80%) did not agree that HoloLens operation required a high level of physical effort (mean 2.27, SD 1.033). This was supported by the low mean score of the items regarding device heaviness (mean 2.53, SD 1.06) and associated fatigue (mean 1.93, SD 1.033). More than half (8/15, 53%) of the mentees agreed that a high level of concentration was required to operate the HoloLens (mean 2.93, SD 1.033).

The mentors reported positively on the mentees’ use of the HoloLens and the AR technology. They reported the ease of use and highlighted the feature of transmitting a live stream from the scene, which helped them to promptly assess the situation and provide guidance. The AR functions, such as annotation or reference insertion, were reported to be well integrated into the AR setup (mean 3.75, SD 0.5). They noted that the AR headset performed well even when the mentee was performing the physically intense activity of cardiopulmonary resuscitation.

All mentees and mentors were satisfied with the interaction with the HoloLens and AR setup, despite several user-related technical issues in using the HoloLens being revealed during the postassessments of the video recordings. Incorrect hand gestures were the cause of a range of accidents in most of the simulation sessions. The issue was the device becoming overheated or shutting down automatically. These issues resulted in >112 minutes of delay in the 21 scenarios.

The mentees were satisfied with the display of the HoloLens (mean 4.2, SD 0.561) and commented that overall, it provided good visual information essential for assessing the clinical situation. However, mentors noted that the small print size on medication vials and entries on patient charts were sometimes blurry and difficult to read. This was compensated by additional audio communication being initiated by the mentor with the mentee.

Participants reported that the scenarios were realistic and that they were satisfied with the fidelity of the simulations and the usability of the HoloLens. Mentors found that the AR technology immersed them in the scenarios. They perceived AR technology as an effective way to provide situational mentorship in other urgent clinical scenarios.

In aged care in Tasmania, often we don’t have doctors or experienced nurses on-site, having something like HoloLens will be very helpful when our senior residents need urgent reviews (e.g., cellulitis, pneumonia, falls). Not to mention our ramping ambulance service, the paramedics often could not attend the facility quick enough. We might be able to contact a GP (General Practitioner) via HoloLens, and the GP may be able to complete an initial assessment and escalate the case immediately if indicated.

Mentee 14

### Mentorship Effectiveness

Despite the first meeting being in the simulation session, the mentees and mentors commented positively about each other and their professional relationship in general. The positive results were also reported statistically in most of the items in the scales of mentorship effectiveness for mentees (13 items), as shown in Table 2, and mentors (6 items), as shown in Table 3. The 5-point Likert scale, with 1 for “strongly disagree” and 5 for “strongly agree,” was also used.

The relationship usually started with the mentee’s needs. The mentee called the mentor once they encountered difficulty with patient management. Depending on the mentee’s capability, the mentor was flexible in assisting them. Some examples of the mentor’s assistance in practice were pointing out things the mentees were unaware of, ensuring they did not skip any steps, correcting medications, and interpreting patient examination results. The flexibility of the mentee’s need-based approach in guidance delivery allowed the mentees to self-lead while being supported via the AR device.

Overall, the satisfaction of both the mentees and mentors was high, with mean scores of 4.8 (SD 0.41) and 4.25 (SD 0.50) out of 5, respectively. The response and expertise of the mentors were highly acknowledged by the mentees, with mean scores of 4.73 (SD 0.458) and 4.53 (SD 0.64), respectively. The mentees felt that the mentors demonstrated their professional integrity well (mean 4.47, SD 0.743), whereas the mentors believed that the mentees matched well to their skills and experience (mean 3.75, SD 0.5). The mentees also highly rated the mentors’ support and encouragement, with a mean score of 4.87 (SD 0.352).

**Table 2:** Mentorship effectiveness scale for mentees (n=15).

Item	Values, mean (SD; range)
My mentor was difficult to communicate with^a^	1.33 (0.488; 1-2)
My mentor demonstrated professional integrity	4.47 (0.743; 3-5)
My mentor demonstrated content expertise in my area of need	4.53 (0.64; 3-5)
My mentor was responsive to my needs	4.73 (0.458; 4-5)
My mentor was supportive and encouraging	4.87 (0.352; 4-5)
My mentor provided constructive and useful critiques of my work	4.53 (0.64; 3-5)
My mentor motivated me to improve my work	4.53 (0.64; 3-5)
My mentor was helpful in providing direction and guidance	4.73 (0.594; 3-5)
My mentor answered my questions satisfactorily	4.73 (0.594; 3-5)
My mentor acknowledged my contributions appropriately	4.67 (0.617; 3-5)
My mentor suggested appropriate resources	4.47 (0.743; 3-5)
My mentor challenged me to extend my abilities	3.8 (1.082; 2-5)
Overall, I was satisfied with my mentor	4.8 (0.414; 4-5)

^a^The items were reverse-coded when calculating the overall mean.

**Table 3:** Mentorship effectiveness scale for mentors (n=4).

Item	Values, mean (SD; range)
My mentees were well-matched to my skills and experience	3.75 (0.5; 3-4)
My mentees were difficult to communicate with^a^	2 (0.816; 1-3)
I was able to answer my mentees’ questions satisfactorily	4.25 (0.5; 4-5)
I was helpful in providing direction and guidance to my mentees	4 (0; 4-4)
I have had a positive impact on my mentees’ performance	3.75 (0.5; 3-4)
Overall, I was very satisfied with the mentoring relationship	4.25 (0.5; 4-5)

^a^The items were reverse-coded when calculating the overall mean.

### Self-Confidence

There are a total of 19 clinical skills in ACS, 23 in AMI, 19 in PSRA, and 23 in HE required to be completed in the simulation sessions. These clinical skills comprised 5 practical skill groups: examination preparation, patient physical examination, communication with the patient, clinical interventional procedures, and medication administration.

The mentees appeared nervous and less confident in all 4 scenarios at the beginning. Analysis of the responses to the self-confidence scale revealed that the mean score of general confidence was highest in the AMI scenario (2.73, SD 0.458) but still under the medium confidence level (3) on the 5-point Likert scale, with 1 for “no confidence at all” and 5 for “very high confidence.” The level of self-confidence was lowest in the medication administration skill group in all 4 scenarios, with medians ranging from 3.00 (AMI [IQR 2.50-3.25] and PSRA [IQR 2.20-3.40]) to 3.40 (ACS [IQR 3.00-4.00]).

The mentees appeared significantly more confident in the simulation environment and in using the AR technology immediately after each scenario performance (all *P*>0.5). The median posttest scores in general confidence were at a high level (4.00, IQR 3.00-4.00) in all the scenarios.

The mean scores before and after the simulation sessions revealed a clear improvement in the mentees’ confidence levels after being mentored using the AR setup. The improvement occurred in all practical skills including those the mentees performed by themselves before the call (ie, washing hands, identifying the patient, introducing themself, and asking the patient for consent) and under observation or remote instruction via the HoloLens during the call. These data were subjected to the Wilcoxon signed rank test, with the results showing statistically significant gains in all skill groups in all 4 scenarios (*P*<.001; Table 4).

**Table 4:** Results of the Wilcoxon signed rank test for the self-confidence questionnaire (n=15).

Practical skill group and results			ACS^a^ scenario		AMI^b^ scenario		PSRA^c^ scenario		HE^d^ scenario
**Examination preparation**									
	Presimulation, median (IQR)	4.00 (3.00-4.00)		4.00 (3.00-4.00)		4.00 (3.00-4.50)		3.67 (3.33-4.50)	
	Postsimulation, median (IQR)	4.00 (4.00-5.00)		5.00 (4.00-5.00)		5.00 (4.00-5.00)		4.67 (4.00-5.00)	
	*Z* score	−2.699		−2.831		−2.701		−3.306	
	*P* value	.007		.005		.007		.001	
**Patient physical examination**									
	Presimulation, median (IQR)	3.50 (3.00-3.88)		3.63 (3.00-3.88)		3.29 (3.00-3.71)		4.00 (3.25-4.13)	
	Postsimulation, median (IQR)	4.25 (3.88-4.75)		4.25 (4.00-4.63)		4.00 (3.71-4.43)		4.38 (3.88-4.88)	
	*Z* score	−3.306		−3.307		−3.419		−3.245	
	*P* value	.001		.001		.001		.001	
**Communication with the patient**									
	Presimulation, median (IQR)	3.25 (3.00-3.75)		4.00 (3.67-4.33)		4.00 (3.75-4.25)		3.33 (2.83-3.67)	
	Postsimulation, median (IQR)	4.25 (3.75-4.50)		4.67 (4.33-4.67)		4.50 (4.00-4.75)		4.33 (3.67-4.67)	
	*Z* score	−3.282		−2.858		−2.623		−3.415	
	*P* value	.001		.004		.009		.001	
**Clinical interventional procedures**									
	Presimulation, median (IQR)	N/A^e^		3.17 (3.00-3.83)		3.00 (2.00-3.00)		N/A	
	Postsimulation, median (IQR)	N/A		4.33 (3.83-4.83)		4.00 (3.00-5.00)		N/A	
	*Z* score	N/A		−3.416		−3.272		N/A	
	*P* value	N/A		.001		.001		N/A	
**Medication administration**									
	Presimulation, median (IQR)	3.40 (3.00-4.00)		3.00 (2.50-3.25)		3.00 (2.20-3.40)		3.33 (2.67-3.67)	
	Postsimulation, median (IQR)	4.80 (4.00-5.00)		4.75 (4.00-5.00)		4.20 (3.80-5.00)		4.33 (4.00-5.00)	
	*Z* score	−3.301		−3.414		−3.411		−3.303	
	*P* value	.001		.001		.001		.001	
**Overall confidence**									
	Presimulation, median (IQR)	3.00 (2.00-3.00)		3.00 (2.00-3.00)		2.00 (2.00-3.00)		2.00 (2.00-3.00)	
	Postsimulation, median (IQR)	4.00 (3.00-4.00)		4.00 (3.00-4.00)		4.00 (3.00-4.00)		4.00 (3.00-4.00)	
	*Z* score	−3.314		−3.217		−3.286		−3.145	
	*P* value	.001		.001		.001		.002	

^a^ACS: acute coronary syndrome.

^b^AMI: acute myocardial infarction.

^c^PSRA: pneumonia severe reaction to antibiotics.

^d^HE: hypoglycemic emergency.

^e^N/A: not applicable; owing to no skill in this group.

### Skill Performance

During the simulation sessions, various prompts were used through 60 times of scenario performances with voice (833 times), visual (ie, images or PDF files; 104 times), and annotation (47 times). Regarding the 5 practical skill groups, the mentors used voice and visual prompts the most to instruct the mentees in the patient examination (275 and 41, respectively) and medication administration (241 and 31, respectively). All annotations were applied in the patient examination. The voice and visual prompts were used together 87 times, whereas visual prompts were inserted into the mentees’ view 17 times without explanation. The mentees commented that the usefulness of visual prompting allowed them to extend their practice capability, which would not have been possible without the HoloLens.

To assess the mentees’ skill performance, a checklist was taken from the developed scripts of the clinical scenarios. The score for each item is as follows: 0=“did not perform,” 1=“inaccurately performed,” and 2=“accurately performed.” The average scores of the mentees’ performances in each scenario were 37.31 (SD 1.702) out of 38 (ACS), 44.6 (SD 2.530) out of 46 (AMI), 36.73 (SD 1.624) out of 38 (PSRA), and 44.43 (SD 1.785) out of 46 (HE). Thus, the average of individual performance rates, which are calculated by dividing the average score by the maximum score, ranged from 98% (ACS) to 97% (AMI, PSRA, and HE).

## Discussion

### AR’s Usability

The study recorded the extensive period using HoloLens with all 7 core AR features for remote assistance across all 4 contemporary emergency clinical scenarios. The generally positive perception of mentees and mentors was reported, and technical issues were noted.

From the clinical point of view, the application of AR technology through clinical scenarios provided evidence of its usability far beyond the studies on a single clinical procedure. For instance, the participants in the study by Ingrassia et al [35] used the Holo Basic Life Support and Defibrillation, a HoloLens-based self-instruction training system with a basic life support simulation, to perform a resuscitation procedure for an adult experiencing cardiac arrest only. On the basis of the comparison between the findings of the studies, we assumed that the longer the use period, the higher the confidence level with the technology, the better the willingness to use it again, and the higher the satisfaction with the display quality. This hypothesis supports the argument reported by Chaballout et al [36] that an excessive cognitive load may impair user perceptions and performance and reduce attention and problem-solving skills. This study also observed a higher level of concentration and effort of the mentees to complete their 4 continuous critical scenarios than that found by Ingrassia et al [35]. Despite differences in user perception, both studies found that the HoloLens was easy to use, with similar scores (approximately 4 out of 5 on the Likert scale).

The AR annotation offered by the HoloLens in the AR setup enabled the mentors to provide the mentees with better remote instruction and increased performance. In this study, our mentors used annotation to instruct the mentees on abnormalities on electrocardiograms, chest x-rays, and patient monitors. Such use was slightly different from the investigation by Rojas-Muñoz et al [37], where the mentors used annotations to demonstrate surgical tools, locate anatomical structures, and show the location and length of incisions.

Furthermore, the AR setup features reference insertion and electronic database access, potentially making the HoloLens a daily tool in operating rooms or COVID-19–related clinics when it is vital to keep the surgical theater sterile or limit the risk of virus transmission by minimizing direct contact [38,39]. These features allow the users to interact with web-based documents, such as patient records, laboratory test ordering, or prescribing. In our experiment, the mentors directly inserted 104 images and PDF documents into the mentees’ field of view, equivalent to an average of approximately 1.7 references per scenario. The inserted references were used to support the mentees in informing the patient status, assessing patient conditions, administering medications, and managing patient situations. In parallel, our mentees accessed a simulated patient database 51 times for 12-lead electrocardiograms or chest x-rays. Martin et al [39] also investigated these AR features on the HoloLens 2 and reported that they potentially improved situational awareness, informed better clinical decision-making, and reduced the risk of viral transmission.

Although version 2 of the HoloLens has nearly double the field of view compared with version 1 (54° vs 30° diagonally, respectively), it remains the main limitation contributing to increased cognitive load on the users. The narrow field of view of the device made it difficult for mentors to see the whole scene while mentees were performing clinical procedures on patients. The mentees had to exert more mental and physical effort to compensate for this limitation. This finding is consistent with the findings of Baumeister et al [40] and Ingrassia et al [35]. In the simulation sessions, the mentors sometimes asked the mentees to tilt their heads down to see their actions on the patients. These requirements potentially resulted in the mentees focusing more on adjusting the device or their posture. It distracted them from the clinical tasks and annoyed the mentors observing and assessing the mentees’ performance in real time. As a typical example, 4 (27%) out of 15 mentees began compressions and gave breaths via the masks inaccurately during resuscitation procedures, and their mentors did not notice the error. These errors could potentially lead to patient death in a real scenario.

### Mentorship Effectiveness

The effectiveness of the mentorship was evident statistically. The satisfaction with 2-way communication using AR technology was also highlighted. The AR setup satisfactorily filled the gap in the long physical distance and created the relationship between the mentors and mentees during the simulation sessions.

Findings about the quality of the situational telementorship in this study coincide with those of other studies on long-term relationships in the health care sector. Dimitriadis et al [41] investigated the perception of 137 physicians and 308 medical students of their long-term, one-on-one, and face-to-face mentoring relationships. The physicians’ perception of the mentorship was measured at the end of every semester using the same scale [42] as that adopted in this study. The results showed that both the groups had a similar level of satisfaction, reflected in similar scores on the items of “satisfactory answers to the mentees” and “helpful guidance provision.” The mentee-mentor matching in the study by Dimitriadis et al [41] was slightly better than that in this study, as the students selected their mentors based on the calculated matching profiles instead of the random selection used in this study. In another study, Lee et al [43] evaluated the effects of a 3-month one-on-one mentorship between 24 experienced registered nurses and 34 new nurses in a hospital. The program was well designed, with a strict participant recruitment process, training sessions for mentors, monthly mentee-mentor seminars, and operations at the mentors’ respective wards. The reported scores in assessing the mentees’ satisfaction were similar to those in our study on the mentors’ integrity and trustworthiness, content expertise of the guidance, and mentees’ skill extension.

Our study also found a remarkable disparity in mentor satisfaction compared with other studies. Although all mentors in this study were happy with their mentees, the mentors in the study by Lee et al [43] expressed disappointment in the learning of new nurses, whereas the mentors’ stress because of the clinical performance of new staff reached 48% in the study by Hautala et al [44]. Preparation for the mentors before the mentorships may be the cause. The mentors in our study received extensive training and practice as mentors and mentees in clinical scenarios. Therefore, they experienced what the mentees may encounter, which made it easier to empathize with them during the scenario performance. By contrast, Lee et al [43] revealed that their mentors had no experience with the mentorship program and did not know how to provide support.

### Self-Confidence

The results clearly showed the self-confidence the mentees gained after performing clinical scenarios in the simulation sessions. The statistically significant improvement in their self-confidence reflected that the telementorship using the AR setup could increase confidence, even in those who were already quite confident in skills with which they were familiar. A randomized controlled trial investigating an optical see-through AR head-mounted display reported similar findings [30]. The study compared the surgical residents’ self-confidence scores assessed before and after performing a lower-leg fasciotomy on cadaver models between an experimental group receiving the telementoring via the AR head-mounted display and a control group receiving documentary instruction only. Both groups showed a statistically significant increase in self-confidence scores from before to after the experiment.

The confidence improvement reported via AR-based telementoring systems in this study was consistent with the studies on virtual reality (VR)–based systems or face-to-face training. For example, Chowriappa et al [45] validated robot-assisted surgery skills acquisition using a VR-based module for urethrovesical anastomosis. The participants were randomized to receive hands-on surgical training (HoST)–based urethrovesical anastomosis training or a control group that did not receive HoST. With the HoST, the trainees were immersed in a novel simulation-based environment that augmented an actual surgical procedure within a VR framework and guided them via haptic-enabled prompts during the task. As a result, 75% of the participants believed that the HoST could improve their confidence in conducting an actual intervention [45]. In another example, Jacobs et al [46] measured the pre- and postcourse self-confidence scores of 50 surgeons at different seniority levels who attended a 2-day advanced trauma operative management course. The training included in-person lectures, a cadaver experience, an operative model, and an interactive discussion. The study indicated that the self-confidence of surgeons improved, with all participant groups reaching statistical significance, especially in the group of expert traumatologists, followed by surgical attendings, trauma fellows, and senior surgical residents. In addition, Kuhls et al [47] offered advanced surgical skills for exposure to trauma courses to 79 senior residents and fellows. The participants were taught a standardized rapid exposure of vital structures in the extremities, neck, thorax, abdomen, retroperitoneum, and pelvis using a human cadaver, a course manual, standardized slide presentations, and a brief video demonstration. After the courses, the participants reported significantly improved self-confidence in all body regions, implying higher confidence levels in their practice of trauma care and general surgery operations.

### Skill Performance

Despite the mentees being novices, the remote assistance provided by the mentors via the AR setup supported them to perform accurately the practical skills required, with an average individual performance rate of >96% across the scenarios. The absence of a control group and pre-experimental assessment make this study inconclusive as to whether the AR-based telementoring system improves the performance of practitioners. Other studies have also provided relevant evidence. Recent literature demonstrates that the HoloLens 2 can be successfully used in a medical ward, especially during the COVID-19 pandemic [38,39,48]. Levy et al [48] reported the improved efficiency of the medical ward round (30% shorter) when using the HoloLens 2. Using the device allowed the staff to contribute to a quick ward round while giving them sufficient time to perform their clinical duties. Martin et al [39] also reported that most staff agreed that the device improved the quality of communication within the clinical teams, enabled them to make better clinical decisions, and improved the quality of care. However, such findings could potentially lead to the usability and practicality of the AR technology being overestimated, as it was ready in the clinical facilities and units led by motivated and interested staff. In addition, the deployment in a single facility and a nonblinded and nonrandomized approach may lead to implications for the further applicability of these findings.

This study demonstrates the use of an AR device (HoloLens) in clinical practice, similar to recent AR-related studies [30,39]. It is also the first study to measure in detail the number and type of prompts used by mentors in each simulated scenario. The results indicate a high demand from the mentees for using 2D or 3D visual aids in an AR environment, in addition to voice instruction.

### Limitations

This study has some limitations. Similar to recent AR studies in health care [35,49], the small number of participants with limited professionals makes it difficult to draw significant conclusions about the benefits of the proposed AR setup on mentorship and practical outcomes from this study. Another limitation of this study was the short duration of the training sessions. Owing to limited funding, each mentee was offered only approximately 2 hours of pre-experiment training, which was unlikely to be sufficient. In addition, the absence of a control group in this pragmatic quasi-experimental design worked against the comparison of the operation and effectiveness of the AR setup with other setups or technologies in similar experimental conditions.

### Conclusion and Recommendations

This research addresses the gaps identified within the existing professional support literature, using a pragmatic approach to explore the usability of AR in situational telementorship in managing clinical scenarios. It provides insight into the experience of HoloLens use, contributing to the existing body of AR literature and providing guidance for policy and practice. There are four key findings: (1) mentors’ and mentees’ positive perception and usability of the AR setup, (2) mentors’ and mentees’ positive perception and effective telementorship, (3) significant improvement in self-confidence among mentees, and (4) high individual skill performance ratings of mentees.

On the basis of these findings and the experience of the research team, the following is recommended:

- Further investigations to explore the advantages and disadvantages of the application of AR technology to improve health outcomes, remote assistance, and service delivery.
- Further investigations to explore patients’ perception and acceptability of the AR technology and headsets during a clinical visit, as they are the focus of care delivery.
- Comparison with other telecommunication systems or devices (eg, teleconferencing systems, smartphones, and smart glasses) to determine the actual benefits of AR.
- Considering design standards and licensing requirements for mentors involved in situational telementorship.
- Developing policies and standardized treatment procedures for advanced telecommunication technologies that will ensure patient and staff safety, personal information confidentiality, and management purposes.

## Appendix

Multimedia Appendix 1 Augmented reality usability scales for mentees and mentors.

## Abbreviations

ACS: acute coronary syndrome
AMI: acute myocardial infarction
AMPLE: Allergies, Medications, Past Medical History, Last Meal, and Events Leading to Presentation
AR: augmented reality
HE: hypoglycemic emergency
HoST: hands-on surgical training
PSRA: pneumonia severe reaction to antibiotics
VR: virtual reality

## Data Availability

The data sets generated and analyzed during this study are available from the corresponding author upon reasonable request.
