# Supplementary material for: Usability of Augmented Reality Technology in Situational Telementorship for Managing Clinical Scenarios: Quasi-Experimental Study
Source: JMIR Med Educ. 2023 Oct 2;9:e47228. doi: 10.2196/47228 (PMC10580139; doi:10.2196/47228)
Supplement: Multimedia Appendix 1 [file mededu_v9i1e47228_app1.doc]

Table S1. AR usability scale for mentees (n=15).

| **Item** | **Min** | **Max** | **Mean (SD)** |
| --- | --- | --- | --- |
| **General use of HoloLens** | **2.43** | **4.79** | **3.54 (0.675)** |
| I found HoloLens unnecessarily complex* | 1 | 4 | 2.27 (0.961) |
| I would need the support of a technician to be able to use HoloLens during the scenarios* | 1 | 5 | 3.2 (1.474) |
| Various functions were well Integrated into HoloLens | 1 | 5 | 3.87 (0.99) |
| I experienced a delay in audio and video transmission* | 1 | 5 | 2.27 (1.438) |
| Most people would learn to use HoloLens easily | 2 | 5 | 3.53 (0.915) |
| I felt confident using HoloLens | 2 | 5 | 3.8 (0.775) |
| I needed to learn a lot of things before I use HoloLens* | 1 | 5 | 2.93 (1.163) |
| HoloLens was heavy for wear during the scenarios* | 1 | 4 | 2.53 (1.06) |
| The concentration required to operate HoloLens was high* | 2 | 5 | 2.93 (1.033) |
| The physical effort required to operate HoloLens was high* | 1 | 4 | 2.27 (1.033) |
| Arm and hands/fingers fatigue were high* | 1 | 4 | 1.93 (1.033) |
| Eye fatigue was high* | 1 | 4 | 2.6 (0.986) |
| Head and neck fatigue was high* | 1 | 5 | 2.8 (1.207) |
| Overall, HoloLens was easy to use | 2 | 5 | 4.07 (0.704) |
| **Interaction with HoloLens** | **2.67** | **4.33** | **3.57 (0.423)** |
| I had the right level of control over what I wanted to do | 2 | 5 | 3.93 (0.799) |
| The effect of my interaction with HoloLens was easily anticipated | 2 | 5 | 3.73 (0.799) |
| The HoloLens did not behave as I expected* | 2 | 4 | 2.33 (0.617) |
| I could not always achieve what I wanted HoloLens to do* | 1 | 5 | 3.33 (1.113) |
| I kept making mistakes interacting with HoloLens* | 2 | 4 | 2.87 (0.834) |
| Hand interactions were difficult to perform* | 1 | 4 | 2.53 (0.834) |
| Hand interactions were easily recognised by HoloLens | 2 | 5 | 3.53 (0.99) |
| Overall, the interaction with HoloLens was satisfactory | 3 | 5 | 4 (0.378) |
| **Display of the HoloLens** | **2.89** | **4.89** | **3.90 (0.454)** |
| The content displayed was appropriate for the scenarios | 3 | 5 | 4.33 (0.617) |
| The observable area was too narrow to see the virtual contents and carry out the tasks* | 1 | 4 | 2.33 (0.9) |
| The screen was not clear because of outdoor ambient light, reflection, or glare* | 1 | 4 | 2 (0.756) |
| The display could be seen flickering* | 1 | 4 | 2.4 (0.828) |
| Computer-generated graphics contents were unstable* | 1 | 4 | 2.27 (0.884) |
| The words and symbols on the screen were easy to read | 2 | 5 | 3.67 (0.9) |
| The quality of the display affected my performance* | 1 | 4 | 2.2 (1.014) |
| The audio instructions provided were clear to understand | 2 | 5 | 4.13 (0.834) |
| Overall, the display of the HoloLens was satisfactory | 3 | 5 | 4.2 (0.561) |
| **Situational awareness** | **4** | **5** | **4.67 (0.418)** |
| I felt the mentor was with me at the scene | 4 | 5 | 4.53 (0.516) |
| I felt that my actions were always followed and supported promptly by the mentor | 4 | 5 | 4.73 (.458) |
| Overall, the situation awareness was satisfactory | 4 | 5 | 4.73 (0.458) |
| **Effectiveness** | **3.6** | **5** | **4.44 (0.530)** |
| I could see a real benefit in augmented reality devices like this one | 4 | 5 | 4.6 (0.507) |
| HoloLens could help me to be more effective in a tele-mentoring situation | 3 | 5 | 4.4 (0.737) |
| HoloLens could help me to be more productive | 2 | 5 | 4.4 (0.910) |
| HoloLens did everything I would expect it to do | 3 | 5 | 4.33 (0.724) |
| Overall, HoloLens was effective to use | 4 | 5 | 4.47 (0.516) |
| **Likeability** | **3** | **5** | **4.04 (0.602)** |
| I was comfortable using HoloLens | 3 | 5 | 4.13 (0.516) |
| I would be happy to use HoloLens again | 4 | 5 | 4.6 (0.507 |
| I would have preferred alternative ways to interact with my mentor* | 1 | 4 | 2.6 (1.056) |

* The items were reverse-coded calculating the overall mean.

Table S2. AR usability scale for mentors (n=4).

| **Item** | **Min** | **Max** | **Mean (SD)** |
| --- | --- | --- | --- |
| **General use of the setup** | **3.38** | **4.13** | **3.57 (0.423)** |
| I found the setup unnecessarily complex* | 2 | 2 | 2 (0) |
| I would need the support of a technician to be able to use the setup during the scenarios* | 1 | 5 | 2.75 (1.708) |
| Various functions were well integrated into the setup | 3 | 4 | 3.75 (0.5) |
| I felt a delay in audio and video transmission* | 1 | 4 | 2.25 (1.258) |
| Most people would learn to use the setup easily | 4 | 4 | 4 (0) |
| I felt confident using the setup | 3 | 4 | 3.5 (0.577) |
| I felt dizzy when following the movement of the mentee* | 1 | 2 | 1.75 (0.5) |
| Overall, the setup was easy to use | 4 | 4 | 4 (0) |
| **Interaction with the setup** | **3.33** | **4** | **3.69 (0.306)** |
| I had the right level of control over what I wanted to do | 4 | 4 | 4 (0) |
| The effect of my interaction was easily anticipated | 4 | 4 | 4 (0) |
| The interaction with the SETUP was not as I expected* | 2 | 2 | 2 (0) |
| I could not always achieve what I wanted the SETUP to do* | 2 | 4 | 3 (1.155) |
| I kept making mistakes interacting with the SETUP* | 2 | 3 | 2.5 (0.577) |
| The effect of my interaction was accurately carried out by the SETUP | 2 | 4 | 3.5 (1) |
| The SETUP was responsive | 2 | 4 | 3.5 (1) |
| I would have preferred alternative interaction methods* | 2 | 3 | 2.25 (0.5) |
| Overall, the interaction with the SETUP was satisfactory | 4 | 4 | 4 (0) |
| **Fidelity of simulated scenarios** | **3.5** | **4** | **3.63 (0.250)** |
| The simulated scenarios were realistic | 4 | 4 | 4 (0) |
| I felt disorientated by the augmented reality environment* | 2 | 4 | 2.5 (1) |
| The computer-generated graphics content influenced my performance | 2 | 4 | 3 (1.155) |
| Overall, the fidelity of the simulated scenarios was satisfactory | 4 | 4 | 4 (0) |
| **Engagement with simulated scenarios** | **3** | **4** | **3.56 (0.427)** |
| I felt part of what was happening in the simulated scenarios | 4 | 4 | 4 (0) |
| The quality of the graphics content reduced my sense of presence* | 2 | 4 | 2.5 (1) |
| The limited field-of-view of the display reduced my engagement* | 2 | 4 | 3.25 (0.957) |
| Overall, the engagement with the simulated scenarios was satisfactory | 4 | 4 | 4 (0) |
| **Situational awareness** | **3.67** | **4** | **3.92 (0.167)** |
| I felt I was at the scene with the mentee and simulated patient | 4 | 4 | 4 (0) |
| I received the patient’s response as if I was at the scene | 3 | 4 | 3.75 (0.5) |
| Overall, the situation awareness was satisfactory | 4 | 4 | 4 (0) |
| **Effectiveness** | **4** | **4.6** | **4.35 (0.252)** |
| I could see potential benefits in AR devices like in this SETUP | 4 | 5 | 4.75 (0.5) |
| The SETUP could help me to be more effective in a tele-mentoring situation | 4 | 5 | 4.75 (0.5) |
| The SETUP could help me to be more productive | 4 | 5 | 4.25 (0.5) |
| The SETUP did everything I would expect it to do | 4 | 4 | 4 (0) |
| Overall, the SETUP was effective to use | 4 | 4 | 4 (0) |
| **Likeability** | **3.33** | **4** | **3.83 (0.333)** |
| I was comfortable using the SETUP | 4 | 4 | 4 (0) |
| I would be happy to use the SETUP again | 4 | 4 | 4 (0) |
| I would have preferred alternative ways to interact with my mentee* | 2 | 4 | 2.5 (1) |

* The items were reverse-coded when calculating the overall mean.
